# A NanoBRET-Based H_3_R Conformational Biosensor to Study Real-Time H_3_ Receptor Pharmacology in Cell Membranes and Living Cells

**DOI:** 10.3390/ijms23158211

**Published:** 2022-07-26

**Authors:** Xiaoyuan Ma, Meichun Gao, Henry F. Vischer, Rob Leurs

**Affiliations:** Division of Medicinal Chemistry, Faculty of Science, Amsterdam Institute of Molecular and Life Sciences, Vrije Universiteit Amsterdam, 1081 HZ Amsterdam, The Netherlands; x.ma@vu.nl (X.M.); m.c.gao@vu.nl (M.G.); h.f.vischer@vu.nl (H.F.V.)

**Keywords:** histamine, H_3_R, GPCR, BRET, conformational biosensor

## Abstract

Conformational biosensors to monitor the activation state of G protein-coupled receptors are a useful addition to the molecular pharmacology assay toolbox to characterize ligand efficacy at the level of receptor proteins instead of downstream signaling. We recently reported the initial characterization of a NanoBRET-based conformational histamine H_3_ receptor (H_3_R) biosensor that allowed the detection of both (partial) agonism and inverse agonism on living cells in a microplate reader assay format upon stimulation with H_3_R ligands. In the current study, we have further characterized this H_3_R biosensor on intact cells by monitoring the effect of consecutive ligand injections in time and evaluating its compatibility with photopharmacological ligands that contain a light-sensitive azobenzene moiety for photo-switching. In addition, we have validated the H_3_R biosensor in membrane preparations and found that observed potency values better correlated with binding affinity values that were measured in radioligand competition binding assays on membranes. Hence, the H_3_R conformational biosensor in membranes might be a ready-to-use, high-throughput alternative for radioligand binding assays that in addition can also detect ligand efficacies with comparable values as the intact cell assay.

## 1. Introduction

G protein-coupled receptors (GPCRs) are membrane-associated seven transmembrane (7TM) proteins that trigger intracellular signaling upon binding of extracellular messengers such as hormones and neurotransmitters. GPCR activation by agonists involves conformational changes in the 7TM domain with most significantly an outward movement of the intracellular side of TM6 to create a pocket at the intracellular interface of TM3, TM5, TM6 and intracellular loop (IL)2 to engage the coupling of heterotrimeric G proteins, GPCR kinases, or β-arrestins, as revealed by X-ray crystallography and more recent cryo-electron microscopy (cryo-EM) structures [1,2,3]. This outward movement of TM6 is smaller for partial agonists as compared to full agonists resulting in a sub-efficient coupling of intracellular signaling transducers and consequently submaximal cellular responses despite full receptor occupancy [4,5,6].

Real-time agonist-induced changes in GPCR conformations have been dynamically measured in living cells using intramolecular resonance-energy transfer (RET)-based biosensors by incorporating a RET acceptor molecule into (the truncated) intracellular loop (IL)3 of the GPCR and fusing a RET donor molecule to its C-terminal tail, or vice versa, allowing for the real-time monitoring of the distance between the two GPCR domains [7,8]. In addition, ligand-induced changes in the distance between TM4 and TM6 has also been measured by labeling-introduced cysteines at the intracellular end of these TMs with optimized Cy3B and Cy7 fluorophores followed by single molecule fluorescent RET imaging [6]. In line with structural studies, full agonists induce a larger change in basal RET in these intramolecular biosensors as compared to partial agonists. Moreover, an opposite change of RET can be observed upon the addition of inverse agonists confirming that GPCRs can adopt a conformation with some basal activity in the absence of ligands [9,10,11,12,13]. Initially, cyan and yellow fluorescent proteins (CFP and YFP) were used as fluorescence resonance energy transfer (FRET) donor and acceptor, respectively, in intramolecular GPCR conformation sensors to measure the distance/re-orientation between IL3 and the C-terminal tail, with the substitution of YFP with the much smaller Fluorescein Arsenical Hairpin Binder (FlAsH) as an improved alternative due to its reduced effect on the GPCR structure [14,15,16]. More recently, bioluminescent luciferases such as Renilla luciferase or the engineered NanoLuc in combination with fluorescent proteins, FlAsH, or the self-labeling fluorescent HaloTag have been employed in BRET-based GPCR conformation sensors to allow for the measurements of conformational changes in GPCRs in a microplate reader assay format [11,12,17,18,19,20,21]. 

**Figure 1:**
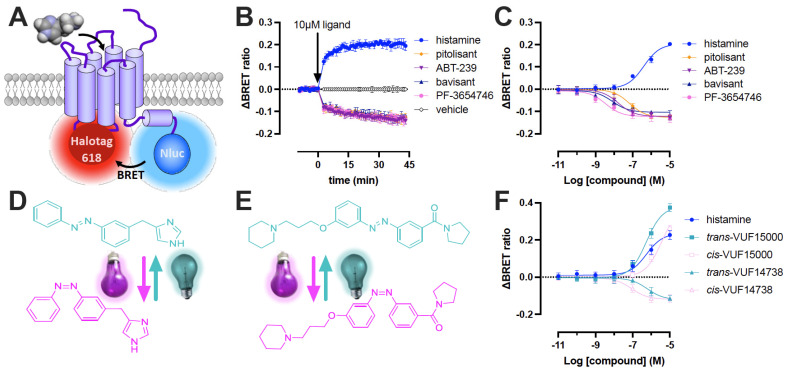
Ligand-induced changes in Δicl3-H_3_R_Nluc/Halo(618)_ biosensor conformation detected by BRET in intact HEK293A cells. (**A**) Scheme of H_3_R biosensor configuration with the self-labeling HaloTag protein inserted in the truncated IL3 between Thr^229^ and Phe^348^ and Nluc fused to the C-terminal tail as the BRET acceptor and donor, respectively. (**B**) Conformational changes in Δicl3-H_3_R_Nluc/Halo(618)_ upon stimulation with 10 μM H_3_R ligands measured as ΔBRET ratio in time. (**C**) Concentration-response curves measured after 30 min stimulation of the H_3_R biosensor with H_3_R ligands. Data are displayed as mean ± SD from 4 independent experiments performed in duplicate. (**D**–**E**) the photo-switchable agonist VUF15000 (**D**) and inverse agonist VUF14738 (**E**) switch from *trans* (cyan) to *cis* (magenta) upon illumination with 360 nm and from *cis* to *trans* by illumination with 430 nm. (**F**) Concentration-response curves measured after 20 min stimulation of the H_3_R biosensor with dark (*trans*) or pre-illuminated (*cis*) photo-switchable VUF15000 and VUF14738. Data are displayed as the mean ± SD from 3 independent experiments performed in duplicate.

We have recently reported on the development and initial characterization of a NanoBRET-based H_3_R conformational biosensor (Δicl3-H_3_R_Nluc/Halo(618)_) that was based on an earlier reported FRET-based H_3_R biosensor with CFP and YFP [22]. In the NanoBRET-based H_3_R sensor the IL3 was substituted from residues Arg^230^ to Arg^347^ with a HaloTag that was subsequently self-labeled with ‘NanoBRET 618’ dye, and NanoLuc was fused in frame to the C-terminal tail (Figure 1A) [12]. The histamine H_3_ receptor (H_3_R) is associated with various neurological disorders such as Alzheimer’s disease, Parkinson’s disease, narcolepsy, and sleeping and learning disorders due to its important role in the central nervous system (CNS) by pre-synaptically controlling the release of histamine and other neurotransmitters including acetylcholine, dopamine, noradrenaline, serotonin, γ-aminobutyric acid, and glutamate [23]. The H_3_R is a constitutively active GPCR that display increased basal signaling in the absence of histamine [24,25]. Moreover, this spontaneous H_3_R activity can be inhibited in native mouse brains by inverse agonists resulting in reduced G protein activation and a consequently increased release of histamine from synaptosomes [26]. Several H_3_R-targeting antagonists/inverse agonists have entered pre-clinical trials for different CNS disorders in the last decade [27,28]. Moreover, pitolisant (Wakix^®^) has been approved as H_3_R inhibitor in 2017 and 2019 by the European Medicine Agency (EMA) and the Food and Drug Administration in the United States (FDA), respectively, to treat patients with narcolepsy [29,30]. 

The new NanoBRET-based H_3_R conformational biosensor accurately discriminates between H_3_R ligands with different efficacies, including full and partial agonists but also inverse agonists, suggesting that it adopts a constitutive active conformation in the absence of ligands [12]. In this study, we have used the Δicl3-H_3_R_Nluc/Halo(618)_ biosensor to pharmacologically characterize a small selection of pre-clinical H_3_R antagonists/inverse agonists and two recently reported photo-switchable H_3_R tool ligands on living cells [31,32]. In addition, we have explored for the first time the function of a GPCR conformational biosensor in membrane preparations instead of intact cells to potentially further increase the assay’s flexibility and throughput.

## 2. Results

### 2.1. Efficacy of (Pre) Clinical and Photoswitchable H_3_R Ligands on the H_3_R Biosensor in Living Cells

Stimulation of HEK293A cells stably expressing the Δicl3-H_3_R_Nluc/Halo(618)_ conformational biosensor with 10 µM of the endogenous agonist histamine or the EMA/FDA-approved inverse agonist pitolisant rapidly increased and decreased BRET, respectively, as compared to the ligand-free (basal) BRET signal (Figure 1B) [12]. The oppositely directed BRET changes both stabilized within approximately 30–45 min. Next, the pre-clinical H_3_R antagonists/inverse agonists ABT-239, PF-3654746 and bavisant were tested on the H_3_R biosensor. All three ligands (10 µM) acted as inverse agonists and reduced the BRET signal with comparable kinetics to pitolisant (Figure 1B), but displayed 4- to 20-fold higher potencies (pEC_50_) than pitolisant (Figure 1C; Table 1), which is in line with their 3- to 25-fold higher binding affinities (pK_i_) for the H_3_R biosensor as compared to pitolisant (Table 1). Bavisant acted as a partial inverse agonist (IA = (−)0.77 ± 0.07) in comparison to pitolisant, whereas both ABT-239 and PF-3654746 (IA = (−)0.96 ± 0.05 and (−)0.92 ± 0.11, respectively) acted as full inverse agonists on the H_3_R biosensor (Figure 1C; Table 1). 

Next, the compatibility of the BRET-based H_3_R biosensor with azobenzene-containing photo-switchable ligands was evaluated. The previously reported photo-switchable H_3_R tool compounds, the agonist VUF15000 and antagonist VUF14738 (Figure 1D–F), showed decreased (*cis*-off) and increased (*cis*-on) binding affinities for the wild type H_3_R upon photo-switching from *trans* into the PSS-*cis* isomer by illumination at 365 nm [31,32]. These affinity shifts were readily translated into a shifted potency (pEC_50_) or antagonizing potency (pIC_50_), respectively, in functional H_3_R assays such as [^35^S]-GTPγS binding to activated G proteins and downstream G protein-coupled inwardly rectifying potassium (GIRK) channel activity [31,32]. First, binding affinities for the photo-switchable ligands were determined in a competition binding assay with [^3^H]NAMH on cell membranes expressing the Δicl3-H_3_R_Nluc/Halo(618)_. The Photo-switchable agonist VUF15000 and antagonist VUF14738 displayed an 8.0-fold decrease and a 31.6-fold increase in binding affinity for the Δicl3-H_3_R_Nluc/Halo(618)_ sensor, respectively, upon photoisomerization from *trans-* to PSS-*cis*-isomer (Table 1), which is comparable to their light-induced affinity shifts on wild type H_3_R (Supplementary Table S1) [31,32]. 

Next, intact cells expressing the H_3_R biosensor were first incubated for 20 min with increasing concentration of the *trans*- or *cis*-isomers of VUF15000 and VUF14738 in the dark, followed by addition of the NanoGlo substrate and immediate detection of ΔBRET signal. Both *trans*- and *cis*-isomers of VUF15000 act as full agonists with higher intrinsic activities than histamine, whereas similar maximum responses were previously observed in a [^35^S]-GTPγS binding [31]. *cis*-VUF15000 displayed a 7.9-fold lower potency as compared to *trans*-VUF15000 (Figure 1F; Table 1). Oppositely, both VUF14738 isomers behave as inverse agonists with *cis*-VUF14738 having a 7.9-fold higher potency than *trans*-VUF14738 (Figure 1F; Table 1). The smaller light-induced shifts in pEC_50_ as compared to pK_i_ values for VUF14738 might be the consequence of unintended *cis* to *trans* switching at 430 nm by the lower wavelength shoulder of the Nluc peak bioluminescence at 460 nm [33]. Hence, the use of red-shifted Nanoluc substrates in combination with far-red acceptor fluophores could be explored in future optimizations of the H_3_R biosensor for photopharmacology research to avoid interference with photoligand switching [34].

### 2.2. Dynamics of H_3_R Biosensor in Intact Cells

**Figure 2:**
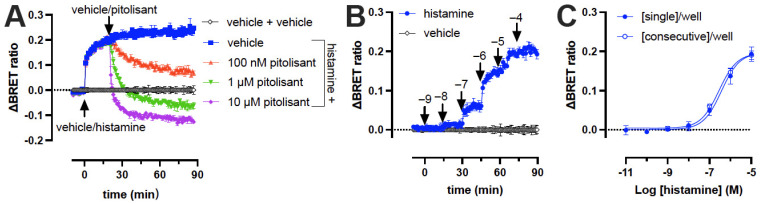
Dynamic changes in Δicl3-H_3_R_Nluc/Halo(618)_ biosensor conformation detected by BRET in intact HEK293A cells. (**A**) Injection of different concentrations pitolisant attenuates the histamine-induced (10 μM) conformational change in the H_3_R biosensor. (**B**) Consecutive injection of increasing (log) concentrations of histamine in the same three wells. Data are displayed as mean ± SD from one representative experiment performed in triplicate. (**C**) Concentration-response curve of histamine generated from Figure 2B, 15 min after each consecutive injection of increasing concentrations histamine in triplicate (3 wells/exp) or 15 min after stimulation of individual wells with increasing concentrations histamine in triplicate (21 wells/exp). Data are displayed as mean ± SD from 3 independent experiments performed in triplicate.

To further explore the dynamics of monitoring conformational changes in the H_3_R biosensor, we first stimulated Δicl3-H_3_R_Nluc/Halo(618)_-expressing cells with 10 µM histamine followed by a second injection after 20 min with vehicle or the competitive inverse agonist pitolisant (0.1 to 10 µM). Pitolisant rapidly antagonized the histamine-induced conformational change of the Δicl3-H_3_R_Nluc/Halo(618)_ biosensor in a concentration-dependent manner and stabilized a more inactive receptor conformation at 1 and 10 µM as compared to vehicle (only)-stimulated cells, indicating that pitolisant fully displaced histamine from the biosensor within the measured timeframe at these concentrations (Figure 2A). In addition, stimulation of Δicl3-H_3_R_Nluc/Halo(618)_-expressing cells by consecutive injections of increasing concentrations of histamine in the same well with 15 min time intervals resulted in a concentration-dependent increase in BRET (Figure 2B). The concentration-response curve (pEC_50_ = 6.6 ± 0.07) generated from the ΔBRET ratios that were taken 15 min after each consecutive injection had a comparable amplitude to the concentration-response curve that was obtained from wells that were each stimulated with a different histamine concentration (pEC_50_ = 6.4 ± 0.1) (Figure 2C).

### 2.3. Behavior of the H_3_R Conformational Biosensor in Membrane Preparations

**Figure 3:**
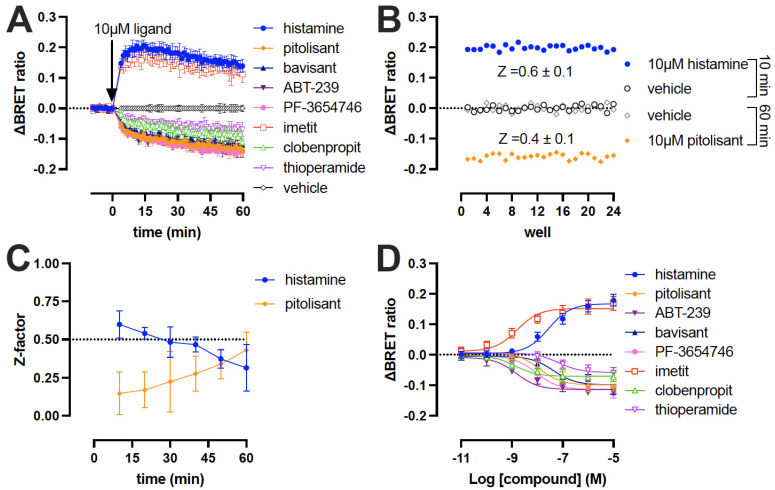
BRET responses of H_3_R ligands determined on Δicl3-H_3_R_Nluc/Halo(618)_ cell membrane prepared in 50 mM Tris-HCl (pH 7.4). (**A**) ΔBRET time course of eight H_3_R ligands at 10 μM concentration. (**B**) ΔBRET ratio measurements in 24 wells of a 96-well plate containing H_3_R biosensor-expressing cell membranes treated with either vehicle (10 and 60 min), 10 μM histamine (10 min) or 10 μM pitolisant (60 min) to calculate the Z-factor. One representative graph from three independent experiments is shown. (**C**) Z-factors over time of cell membrane treated with 10 μM histamine or pitolisant in 96-well plate. (**D**) Concentration-response curves measured after 30 min stimulation of H_3_R biosensor-expressing membranes with H_3_R ligands. Data are displayed as mean ± SD from at least 3 independent experiments performed in duplicate.

We have previously shown in radioligand binding experiments on cell membrane preparations that the Δicl3-H_3_R_Nluc/Halo(618)_ conformational biosensor binds ligands with comparable affinities to wild type H_3_R [12]. To evaluate whether the conformational biosensor can also detect ligand efficacy, as ΔBRET changes in membrane preparations, the H_3_R biosensor was first labeled with the HaloTag 618 dye, followed by the addition of NanoGlo^®^ substrate and stimulation with a small selection H_3_R ligands that have also been (previously) tested on intact cells. The agonists histamine and imetit (10 μM) induced an increase in the ΔBRET ratio that peaked 15–20 min after stimulation followed by a gradual decrease (Figure 3A), which contrasts with the (previously) observed steady-state response for at least 45 min on intact cells expressing this H_3_R conformational sensor (see Figure 1B) [12]. The agonist peak response in membranes, however, is comparable with the observed steady-state amplitude in intact cells. All tested the inverse agonists (10 μM) steadily reduced the basal ΔBRET signal without reaching a clear steady-state plateau within the 1 h detection timeframe (Figure 3A), whereas stable bottom plateaus were previously observed on intact cells after approximately 30–45 min ligand stimulation (see Figure 1B) [12]. 

The Z-factor for the agonist (10 μM histamine) ΔBRET window was 0.6 ± 0.1 after 10 min stimulation (i.e., peak response) and remained above the required Z ≥ 0.5 up to 40 min indicating that the H_3_R biosensor in membranes is suitable for agonist screening within this timeframe [35]. However, the Z-factor decreased to 0.3 ± 0.15 after 60 min (Figure 3B,C). In contrast, the Z-factor gradually increased over time for the inverse agonist (10 μM pitolisant) ΔBRET window to Z = 0.4 ± 0.1 after 60 min and consequently did not qualify as a useful screening assay within the tested timeframe (Figure 3B,C). Extrapolation of the observed Z-factor over time suggests that a longer incubation period (e.g., 90 min) is required for inverse agonist screening to obtain Z-factors ≥ 0.5 (Figure 3C). Consequently, the simultaneous detection of agonist/inverse agonist-induced conformational changes will not be possible at one particular time-point in an end-point screening format using membranes.

**Figure 4:**
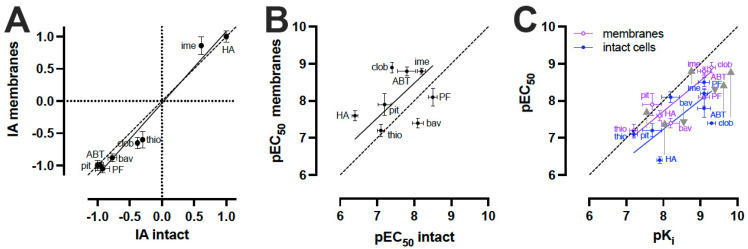
Comparison of H_3_R biosensor pharmacology on intact cells versus membrane preparations in response to H_3_R ligands. (**A**,**B**) Comparison of intrinsic activity (IA) values (**A**) and pEC_50_ (**B**) obtained from H_3_R biosensor in intact cells versus membrane preparations upon stimulation with increasing ligand concentrations for 30 min (see Figure 1C and Figure 3D; Table 1). (**C**) Comparison of pK_i_ values obtained from radioligand competition binding experiments on H_3_R biosensor-expressing membranes in 50 mM Tris-HCl (pH 7.4) with pEC_50_ values obtained from H_3_R biosensors in intact cells (in HBSS) and membrane preparations (in 50 mM Tris-HCl (pH 7.4)) upon stimulation with increasing ligand concentrations for 30 min (see Figure 1C and Figure 3D; Table 1). Differences between pEC_50_ values obtained from H_3_R biosensor in intact cells versus membrane preparations are indicated with grey arrows. Data are displayed as mean ± SD from at least 3 independent experiments performed in duplicate. Deming linear regression was used to compare the fitted affinity and/or potency values between the different assay formats, the dotted line represents line of unity (**B**,**C**). HA = histamine; ime = imetit; pit = pitolisant; clob = clobenpropit; thio = thioperamide; bav = bavisant; ABT = ABT-239; PF = PF-3654746.

Full concentration-response curves on the H_3_R biosensor in membranes were measured 30 min after stimulation with H_3_R agonists and inverse agonists (Figure 3D), resulting in intrinsic activity values that were largely comparable to those observed in intact cells (Figure 4A). Relative to the reference ligands histamine (IA = 1) and pitolisant (IA = −1), agonist imetit and all tested inverse agonists seemed to have a slightly increased amplitude (IA) on the H_3_R biosensor membranes as compared to intact cells (Figure 4A; Table 1). 

Although some correlation was observed for the pEC_50_ values on the H_3_R biosensor in membranes versus intact cells, the rank order was different (membranes: thioperamide < bavisant < histamine <pitolisant < PF-3654746 < imetit < ABT-239 < clobenpropit versus intact cells: histamine < thioperamide <pitolisant <clobenpropit < ABT-239 < bavisant <imetit < PF-3654746) (Figure 4B; Table 1). Remarkably, histamine and clobenpropit showed a 16- and 32-fold higher potency, respectively, to change the H_3_R biosensor conformation in membranes preparations as compared to intact cells, whereas ABT-239 was 10-fold more potent on membrane preparations. Smaller potency differences (<5-fold) were observed for the other tested ligands with slightly increased potencies for pitolisant and imetit on H_3_R biosensor-expressing membranes but with decreased potency values for bavisant and PF-3654746. The potency of thioperamide was not significantly different between intact cells and membrane preparations. 

One explanation for these observed potency differences is that ligand-induced H_3_R biosensor conformational changes were measured in two different buffers between membranes and intact cells, i.e., 50 mM Tris-HCl (pH 7.4) versus HBSS, respectively, and binding affinities for at least some H_3_R ligands are known to be considerably different between buffers that contain different salt concentrations [36,37,38]. Indeed, pEC_50_ values measured on intact cells expressing the H_3_R biosensor in HBSS containing 138 mM NaCl were lower for all the tested ligands, except for thioperamide, as compared to their pK_i_ values measured on H_3_R biosensor-expressing membranes in 50 mM Tris-HCl buffer (pH 7.4) (Figure 4C; Table 1). Measuring ligand binding and conformational H_3_R changes on membranes in the same 50 mM Tris-HCl buffer (pH 7.4) yielded a better correlation between binding affinities and potency values for most ligands, except for bavisant and PF-3654746.

## 3. Discussion

Detection of conformational changes in GPCRs using RET between donor and acceptor molecules that are inserted in between TM5/TM6 and the C-terminal tail allows for the direct quantification of agonist and inverse agonist potency and efficacy upon ligand binding to the receptor. The FRET-based H_3_R sensor in intact cells and cultured on cover slips allowed for the rapid detection of ligand-induced conformational receptor changes using a fluorescent microscope equipped with a perfusion system with high temporal resolution but a relatively low throughput [22]. Substitution of YFP and CFP with respectively a red fluorescent dye covalently bound to HaloTag and NanoLuc allows a NanoBRET-based detection of conformational H_3_R changes in living cells using a 96-well plate reader-based format to readily generate full concentration-response curves for multiple ligands in a single assay run [12], as also previously optimized and reported for the α_2A_-adrenergic receptor, β_2_-adrenergic receptor, and the parathyroid hormone 1 receptor [11,21]. As a follow-up on our initial report on this sensor, we evaluated a number of well-known H_3_R tools (photo-switchable ligands or preclinical candidates) for their conformational effects. Our data indicate that all the preclinical candidates indeed act as inverse agonists, with bavisant showing a clear partial inverse agonistic effect. Moreover, the sensor also allowed the evaluation of the recently developed photo-switchable agonist and antagonist [31,32], although the light generated by the NanoLuc donor might to some extent also affect the *cis-trans* ratio due to spectral overlap of the *cis*-isomer and the NanoLuc emission.

Although this 96-well assay format significantly increases the ligand screening throughput, most microplate readers are only equipped with one or two injectors and consequently do not allow much flexibility with respect to adding multiple ligands and/or concentrations during BRET measurements. In this study we show that measurements can be paused to remove the plate from microplate reader to manually add (consecutive) ligands to the assay plate and continue the readout. Indeed, the rapid addition of pitolisant to cells that were pretreated with histamine resulted in a concentration-dependent decrease of the BRET signal showing that histamine can be quickly displaced from H_3_R by pitolisant thereby switching the receptor from an active into an inactive conformation. This is in line with the complete dissociation of histamine from the FRET-based H_3_R sensor within approximately 15 s upon washout [22]. Such a washout experiment is difficult to repeat using the NanoBRET-based H_3_R sensor as this will also remove the NanoGlo substrate resulting in reduced reproducibility, which could not be easily restored even by supplementing fresh NanoGlo. 

In contrast to G protein-mediated downstream signaling assays, the agonist concentration–response curves on a conformational GPCR are not subjected to signal amplification and the observed potency should be comparable to binding affinity of the ligand for the receptor [39]. This makes the NanoBRET-based H_3_R biosensor very useful for initial drug discovery as a measure for ligand affinity and efficacy can be simultaneously obtained. However, performing pharmacological assays on living cells requires the constant availability of cells in their exponential growth phase, which can limit the numbers of assays. Considering that the NanoBRET-based H_3_R biosensor displayed comparable binding affinities for all tested ligands as the wild type H_3_R in membrane preparations that were generated from frozen cell pellets, we decided to evaluate the ligand-induced conformational H_3_R changes in these membranes. Ligand potency (pEC_50_) values measured on membranes were more in line with affinity values (pK_i_) obtained from radioligand competition binding assays, as compared to the potencies measured on intact cells. This is most likely related to presence of NaCl in the HBSS medium that is used for the intact cell assay, and known to affect binding affinities of H_3_R ligands [36,37,38]. Yet, a good correlation between the intrinsic activity of both agonists and inverse agonists was observed between the intact and membrane H_3_R conformational sensor assays.

In conclusion, the H_3_R biosensor in membranes could be a useful alternative for radioligand binding assays and allows for the simultaneous measurement of ligand affinity (via its potency) and efficacy on the H_3_R. Moreover, membranes expressing the H_3_R biosensor can be prepared in a large batch and stored in the freezer as (nearly) ready-to-use cell pellets to avoid prolonged and time-consuming culturing of an H_3_R biosensor-expressing stable cell line that is required for living cell assays.

## 4. Materials and Methods

### 4.1. Materials

Fetal bovine serum was obtained from Bodinco (Alkmaar, The Netherlands), and penicillin/streptomycin was purchased from GE Healthcare (Uppsala, Sweden). Dulbecco’s Modified Eagles Medium (DMEM, #41966-029), Dulbecco’s phosphate-buffered saline (DPBS, #D8662), trypsin-EDTA and Hanks’ Balanced Salt Solution (HBSS, #14025-050) were bought from Thermo Fisher Scientific (Waltham, MA, USA). Geneticin was obtained from Sigma-Aldrich (Taufkirchen, Germany). Linear polyethylenimine (PEI, 25-kDa) was obtained from Polysciences (Warrington, PA, USA). N^α^-[*methyl*-^3^H] histamine ([^3^H]NAMH) (specific activity 81.7 Ci/mmol), Microscint-O scintillation liquid, GF/C filter plates and a Microbeta Wallac Trilux scintillation counter were purchased from PerkinElmer (Groningen, The Netherlands). Histamine·2HCl and imetit·2HBr were bought from Sigma-Aldrich (St. Louis, MO, USA). Thioperamide and clobenpropit were purchased from Abcam (Cambridge, UK), PF-3654746 and ABT-239 were obtained from Axon Medchem (Groningen, The Netherlands), and pitolisant and bavisant (JNJ-31001074) were obtained from Griffin Discoveries (Amsterdam, the Netherlands). VUF15000 and VUF14738 were synthesized in house as described previously [31,32]. NanoGlo^®^ (N1130) and HaloTag^®^ NanoBRET™ 618 Ligand (G9801) were bought from Promega (Madison, WI, USA). All other reagents were of analytical grade and obtained from conventional commercial sources.

### 4.2. Photochemistry

The photo-switchable compounds (VUF15000, VUF14738) were synthesized in-house and their in-depth photochemical properties were previously reported [31,32]. Briefly, both compounds have an λ_max_ value for the π-π* transition of the *trans*-isomer of 360 ± 20 nm and an n-π* transition of the *cis*-isomer of 430 ± 17 nm. Photo-switchable compounds (10 mM in DMSO) were illuminated with 360 ± 20 nm light for 300 s to reach a photostationary state (PSS) containing over 86% of the *cis*-isomer or kept in dark to ensure more than 99% of the *trans*-isomer. The illumination was carried out in cylindrical clear glass vials of 4.5 mL volume, with a typical distance of 2 cm from the light source. All subsequent experimental steps were conducted in the dark or under near-infrared light. Both *cis*-VUF15000 and *cis*-VUF14738 have thermal relaxation half-lives of >100 days at room temperature.

### 4.3. Cell Culture

HEK293A cells stably expressing the Δicl3-H_3_R_Nluc/Halo(618)_ biosensor were cultured in DMEM (Dulbecco’s modified Eagle’s medium) supplemented with 10% FBS, 1% penicillin/streptomycin, 2 mM glutamine and 500 μg/mL geneticin at 37 °C, 5% CO_2_, as previously described [12]. 

### 4.4. BRET-Based H_3_R Biosensor Detection on Intact Cells

Cells were collected in culture medium supplemented with 50 nM HaloTag NanoBRET 618 dye, transferred into white bottom 96-well plates (50,000 cells/well) and cultured for another 24 h. Next, the culture medium was replaced by a 1/1000 dilution of NanoGlo^®^ stock solution in HBSS. Subsequently, ligand solution or vehicle control was added and the stimulated BRET ratio was recorded at 37 °C using a BRETplus1 luminescence module (610 nm and 460 nm) of the PHERAstar FS (BMG labtech GmbH, Ortenberg, Germany). To avoid unintended backswitching of PSS-*cis* into the *trans*-isomer of the photo-switchable ligands VUF15000 and VUF14738 (λ_max_ = 427) by the Nluc luminescent peak emission at 460 nm, the cells were first incubated for 20 min with the photo-switchable tool ligands followed by the addition of NanoGlo^®^ solution and direct luminescence detection at 460 and 610 nm [31,32,33].

### 4.5. Membrane Preparation

HEK293A cells that stably express the Δicl3-H_3_R_Nluc/Halo(618)_ biosensor were collected from 10 cm dishes (90% confluency) as previously described [12]. Briefly, cells were detached using cold phosphate-buffered saline (PBS) and centrifuged at 1900× *g* for 15 min at 4 °C. The supernatant was discarded and the cell pellet was stored at freezer (−20 °C) for further experiments. On the day of the experiment, cell pellets were resuspended (4–6 mL/10 cm dish) in 50 mM Tris-HCl (pH 7.4) and disrupted using a Branson 250 sonifier (Boom B.V., Meppel, The Netherlands).

### 4.6. [^3^H]NAMH Competition Binding Assay on Membranes

Membrane suspensions (50 μL/well) were incubated with 2 nM [^3^H]NAMH in combination with increasing concentrations of unlabeled ligands for 2 h at 25 °C with gentle agitation. Incubation was stopped by harvesting the homogenates onto 96-well GF/C plates pre-soaked with 0.5% (*v*/*v*) PEI using a 96-well Filtermate harvester (PerkinElmer, Groningen, The Netherlands). The GF/C filter plates were then washed three times with cold wash buffer (50 mM Tris-HCl, pH 7.4, 4 °C) and dried for 30 min. Filter-bound radioactivity was quantified by a Microbeta Wallac Trilux scintillation counter (Perkin-Elmer) after addition of 25 μL/well scintillation liquid.

### 4.7. BRET-Based H_3_R Biosensor Detection on Membranes

Membrane suspensions (50 μL/well) were incubated with 50 nM HaloTag NanoBRET 618 dye for 2 h at 25 °C. Next, NanoGlo^®^ stock solution (1/1000 dilution) was added per well and the basal BRET ratio was measured. Subsequently, ligand solution or vehicle control was added, and the stimulated BRET ratio was recorded at 25 °C.

### 4.8. Data Analysis

GraphPad Prism version 9.0 (GraphPad Software, San Diego, CA, USA) was used for data analysis and statistics.

BRET ratios were calculated by dividing the BRET signal at 610 nm by the Nluc signal at 460–480 nm. ΔBRET was used for quantifying ligand-induced changes in BRET ratio using the following equation:

(1) $$\Delta \mathrm{BRET}=\frac{\mathrm{BRET}\;\left[\mathrm{stim}\right]-\mathrm{BRET}\;\left[\mathrm{vehicle}\right]\;}{\mathrm{BRET}\;\left[\mathrm{vehicle}\right]}$$

Concentration-response curves were fitted using the “log (agonist) vs. response (three parameters)” model:

(2) $$\mathrm{response}\;=\mathrm{bottom}\;+\frac{\mathrm{top}\;-\mathrm{bottom}}{1+10^{\left({\mathrm{Log}\;\mathrm{EC}}_{50}-\mathrm{Log}\;\left[A\right]\right)}}$$

Intrinsic activity (IA) value is calculated as:

(3) $$\mathrm{IA}=\frac{\mathrm{fitted}\;\mathrm{maximum}\;\mathrm{response}\;\mathrm{agonist}\;\mathrm{or}\;\mathrm{inverse}\;\mathrm{agonist}}{\mathrm{fitted}\;\mathrm{maximum}\;\mathrm{response}\;\mathrm{histamine}\;\mathrm{or}\;\mathrm{pitolisant}}$$

where agonist and inverse agonists were compared to histamine and pitolisant, respectively, and inverse agonism is indicated by (−). 

Competition binding curves were fitted using the “one-site—Fit log IC_50_” model:

(4) $$\mathrm{binding}\;=\mathrm{bottom}\;+\frac{\mathrm{top}\;-\mathrm{bottom}}{1+10^{\left(\mathrm{Log}\;\left[A\right]-{\mathrm{LogIC}}_{50}\;\right)}}$$

Ligand binding affinities (K_i_) were calculated using the Cheng-Prusoff equation [40]:

(5) $$K_{i}=\frac{{\mathrm{IC}}_{50}}{1+\frac{\left[L\right]}{K_{d}}}$$

where [L] and K_d_ represent the concentration and equilibrium dissociation constant of [^3^H]NAMH, respectively.

The Z-factors were calculated based on the following equation [35]:

(6) $$Z-\mathrm{factor}=1-\frac{\left(3\times \;\sigma \left[\mathrm{compound}\right]+3\times \;\sigma \left[\mathrm{vehicle}\right]\right)}{\left(\mu \left[\mathrm{compound}\right]-\mu \left[\mathrm{vehicle}\right]\right)}$$

where σ represents the standard deviation, μ represents the average respectively.

The correlation graphs were analyzed using the “Deming regression” model.

## Figures and Tables

**Table 1:** Potencies (pEC_50_), intrinsic activities (IA) and binding affinities (pK_i_) of H_3_R ligands on the Δicl3-H_3_R_Nluc/Halo(618)_ biosensor expressed on intact cells and membrane preparations, measured in HBSS and 50 mM Tris-HCl (pH 7.4), respectively. The pK_i_ values were calculated using the Cheng–Prusoff equation from the IC_50_ values determined in competition binding with [^3^H]NAMH (Supplementary Figure S1). Data represent the mean ± SD of (n) experiments. n.d.: not determined.

Ligand	Intact Cells		Membrane Preparations		
	pEC_50_	IA ^1^	pEC_50_	IA ^1^	pK_i_
histamine	6.4 ± 0.1 (3) *	(+)1.00 ± 0.02	7.6 ± 0.1 (6) *	(+)1.00 ± 0.09	7.9 ± 0.1 (3)
pitolisant	7.2 ± 0.2 (4) *	(−)1.00 ± 0.03	7.9 ± 0.3 (3) *	(−)1.00 ± 0.08	7.7 ± 0.3 (3)
ABT-239	7.8 ± 0.3 (4) *	(−)0.96 ± 0.05	8.8 ± 0.1 (3) *	(−)0.99 ± 0.04	9.1 ± 0.2 (3)
bavisant	8.1 ± 0.2 (4) *	(−)0.77 ± 0.07	7.4 ± 0.1 (4) *	(−)0.88 ± 0.06	8.2 ± 0.2 (3)
PF-3654746	8.5 ± 0.1 (4) *	(−)0.92 ± 0.11	8.1 ± 0.2 (4) *	(−)1.05 ± 0.06	9.1 ± 0.2 (3)
*trans*-VUF15000	6.3 ± 0.1 (3) ^2^	(+)1.36 ± 0.05 ^2^	n.d.	n.d.	8.0 ± 0.2 (3)
*cis*-VUF15000	5.4 ± 0.2 (3) ^2^	(+)1.35 ± 0.04 ^2^	n.d.	n.d.	7.2 ± 0.0 (3)
*trans*-VUF14738	6.2 ± 0.2 (3)	n.d. ^4^	n.d.	n.d.	6.2 ± 0.1 (3)
*cis*-VUF14738	7.1 ± 0.2 (3)	n.d. ^4^	n.d.	n.d.	7.7 ± 0.3 (3)
imetit	8.2 ± 0.1 ^3,^*	(+)0.61 ± 0.01	8.8 ± 0.1 (3) *	(+)0.86 ± 0.14	9.1 ± 0.1 ^3^
clobenpropit	7.4 ± 0.0 ^3,^*	(−)0.38 ± 0.01	8.9 ± 0.1 (3) *	(−)0.65 ± 0.09	9.3 ± 0.1 ^3^
thioperamide	7.1 ± 0.1 ^3^	(−)0.30 ± 0.01	7.2 ± 0.2 (3)	(−)0.60 ± 0.13	7.2 ± 0.1 ^3^

^1^ IA was calculated using the fitted ligand-induced window “span” as fraction of full agonist histamine for the agonists (+) or using full inverse agonist pitolisant for the inverse agonists (−). ^2^ Data for VUF15000 photoisomers were fitted using the “log(agonist) vs. response (three parameters)” model with a shared top plateau as curves could not be finished and under the assumption that *trans-*and *cis-*isomers have same efficacy as previously reported in [^35^S]-GTPγS binding experiments to measure G protein activation [31]. ^3^ Potency and affinity values that were previously reported on the H_3_R conformational biosensor [12]. ^4^ Intrinsic activity could not be calculated as the inverse agonist reference pitolisant was not included in the same experiments. * statistical difference (*p* < 0.05) in pEC_50_ values of H_3_R biosensor conformational changes between intact cells and membrane preparation in an unpaired *t*-test.

## Data Availability

Not applicable.

## Supplementary Materials

The following supporting information can be downloaded at: https://www.mdpi.com/article/10.3390/ijms23158211/s1.
