# Supplementary material for: A NanoBRET-Based H3R Conformational Biosensor to Study Real-Time H3 Receptor Pharmacology in Cell Membranes and Living Cells
Source: Int J Mol Sci. 2022 Jul 26;23(15):8211. doi: 10.3390/ijms23158211 (PMC9332000; doi:10.3390/ijms23158211)
Supplement: Supplementary file 1 [file ijms-23-08211-s001.zip › ijms-1839677-supplementary.pdf]

A nanoBRET-based H_3R conformational biosensor to study real-time H_3 receptor pharmacology in cell membranes and living cells.

Xiaoyuan Ma, Meichun Gao, Henry F. Vischer and Rob Leurs

Division of Medicinal Chemistry, Faculty of Science, Amsterdam Institute of Molecular and Life Sciences, Vrije Universiteit Amsterdam, 1081 HZ Amsterdam, The Netherlands

Supplementary information

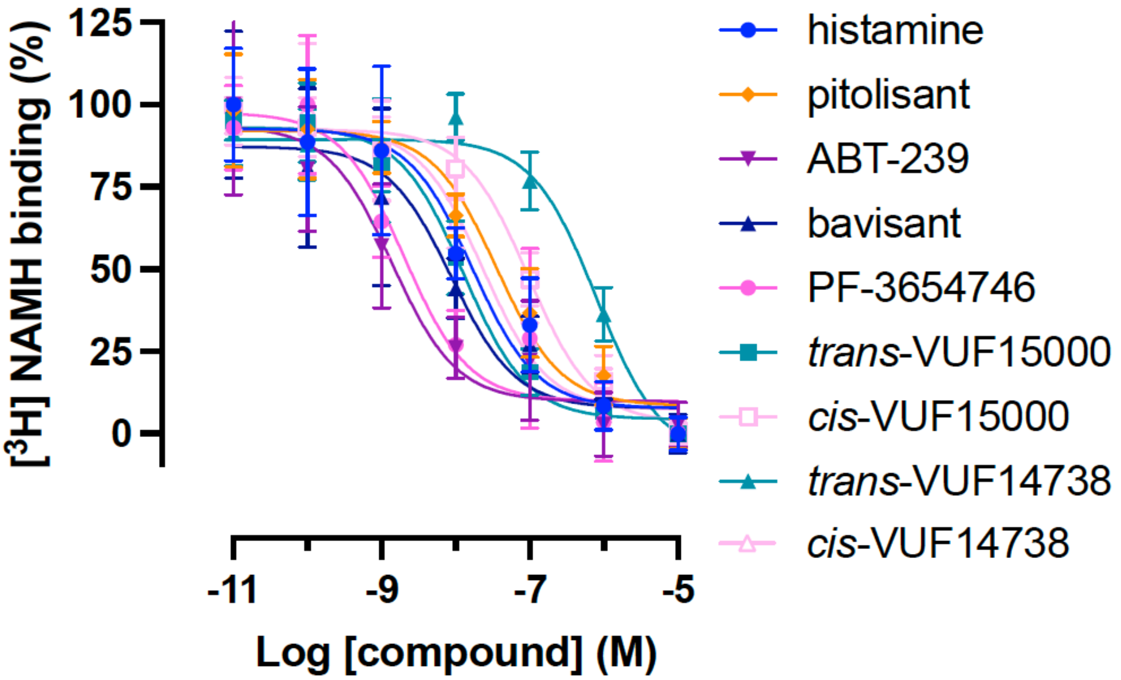

Figure S1. Competition binding of 2 nM [^{3}H]NAMH with increasing concentrations ligands to Δicl3-$H_3R_{Nluc/Halo(618)}$ biosensor-expressing membranes. Data are displayed as mean ± SD from at least 3 independent experiments performed in triplicate.

Table S1. Binding affinity (pK_i) of H_3R photoswitchable compounds for the WT H_3R as determined in competition binding with [^{3}H]NAMH. Data represent the mean ± SD of (n) experiments.

Ligand	WT H_3R
trans-VUF15000	8.2 ± 0.0 (3)
cis-VUF15000	7.4 ± 0.1 (3)
trans-VUF14738	6.5 ± 0.1 (3)
cis-VUF14738	7.3 ± 0.1 (3)
